# Item distribution, internal consistency and inter-rater reliability of the German version of the QUALIDEM for people with mild to severe and very severe dementia

**DOI:** 10.1186/s12877-016-0296-0

**Published:** 2016-06-18

**Authors:** Martin Nikolaus Dichter, Christian G. G. Schwab, Gabriele Meyer, Sabine Bartholomeyczik, Margareta Halek

**Affiliations:** German Center for Neurodegenerative Diseases (DZNE), Witten, Germany; School of Nursing Science, Witten/Herdecke University, Witten, Germany; Institute for Health and Nursing Science, Medical Faculty, Martin Luther University, Halle-Wittenberg, Germany

**Keywords:** Quality of life, Nursing homes, Dementia, Psychometric properties, Reliability

## Abstract

**Background:**

The QUALIDEM is a dementia-specific Quality of life (Qol) instrument that is recommended for longitudinal studies and advanced stages of dementia. Our study aimed to develop a user guide for the German version of the QUALIDEM and to determine the item distribution, internal consistency and inter-rater reliability (IRR) of the German QUALIDEM.

**Methods:**

A user guide was developed based on cognitive interviews with ten professional caregivers and a focus group with six professional caregivers. The item distribution, internal consistency and IRR were evaluated through a field test including *n* = 55 (mild to severe dementia) and *n* = 36 (very severe dementia) residents from nine nursing homes. Individuals with dementia were assessed four times by blinded proxy raters.

**Results:**

A user guide with instructions for the application of the QUALIDEM and definitions and examples for each item was created. Based on the single-measure intra-class correlation coefficient (ICC for absolute agreement), we observed strong IRR for nearly all of the QUALIDEM subscales, with ICCs of at least 0.79. A lower ICC (ICC = 0.64) was only obtained for people with very severe dementia on the ‘negative affect’ subscale.

**Conclusions:**

The IRR improved based on the application of the QUALIDEM user guide developed in this study. We demonstrated a sufficient IRR for all subscales of the German version of the QUALIDEM, with the exception of the ‘negative affect’ subscale in the subsample of people with very severe dementia. The item distribution and internal consistency results highlight the need to develop new informative items for some subscales.

**Electronic supplementary material:**

The online version of this article (doi:10.1186/s12877-016-0296-0) contains supplementary material, which is available to authorized users.

## Background

Health care research that focuses on person-centered outcomes (e.g., Quality of life), particularly for dementia as a chronic and currently incurable syndrome, is an international priority [1, 2]. Therefore, ensuring quality of life (Qol) is a major goal of dementia care [3] and research [4]. The World Health Organization defines Qol as “individuals’ perceptions of their position in life in the context of their culture and value systems in which they live and in relation to their goals, expectations, standards and concerns” [5]. This broad definition focuses on subjective experience, culture-specific influence and their interaction. Subjectivity and multidimensionality are the common denominators in definitions of dementia-specific Qol [6]. This vague definition has influenced the creation of multiple dementia-specific Qol instruments with heterogeneous interpretations of the concept. Some instruments specifically consist of items that assess functional and cognitive abilities, whereas other instruments focus on psycho-social domains of Qol [7]. Self-rating of Qol is regarded as the gold standard [8]. However, for assessing advanced stages of the disease, Qol ratings from a proxy perspective are recommended [4]. Proxy ratings are accompanied by methodological challenges, and the results are systematically lower than those for self-rated Qol [9]. Such ratings are positively correlated with raters’ attitudes [10], burdens [11] and life satisfaction [12]. Probably because of differences between the self and proxy perspectives, demonstrating positive effects on Qol for people with dementia has not been possible for most non-pharmacological interventions [13]. One frequently used instrument for people with dementia living in nursing homes is the QUALIDEM [14], which focuses on psycho-social domains of Qol and is based on the idea that Qol is a result of adaptation to the consequences of the disease. The QUALIDEM is a research instrument that is recommended for application in longitudinal studies [4, 15] and can be used throughout the entire course of dementia via its two consecutive versions: one for people with mild to severe dementia (37 items) and the other for individuals with very severe dementia (18 items) [14]. The QUALIDEM is the only dementia-specific instrument that enables assessment of the Qol domains of ‘care relationship’ and ‘feeling at home’. Both domains are important for people with dementia who live in nursing homes. In 2008, both QUALIDEM versions were translated into German [16]. The psychometric qualities of the original Dutch [14, 17, 18] and German versions of the QUALIDEM [16, 19, 20] have been examined in several studies with positive results. However, the effort required to apply the QUALIDEM in observational and intervention studies is quite high because acceptable results for inter-rater reliability (IRR) can be only obtained by a collaboratively QUALIDEM rating of at least two nurses serving as proxy raters [14, 19].

The first aim of our study was the development of a user guide for the German version of the QUALIDEM that includes detailed definitions and examples for each QUALIDEM item. The second aim was the determination of the item distribution, internal consistency and IRR of the German QUALIDEM based on the application of the new user guide. Due to the application of the user guide, we expected a pronounced improvement of the inter-rater reliability properties of the German version of the QUALIDEM which allow the QUALIDEM application by one caregiver in future studies.

Such an improvement would lead to a reduction in the effort required to obtain acceptable IRR results.

## Methods

### Study design

The study was conducted between May 2014 and February 2015. The user guide was developed based on individual- and focus group-based cognitive interviews. Subsequently, the item distribution, internal consistency and IRR were evaluated in a cross-sectional field test. The IRR of the QUALIDEM was assessed four times by blinded proxy raters.

### Setting and sample

#### Development of the QUALIDEM user guide

Cognitive interviews were conducted with a purposeful sample. We aimed for a sample representing different professional caregiver qualification levels (registered nurses and nursing aids), caregivers with and without experience in the application of the QUALIDEM before the administration of the cognitive interviews and caregivers with and without a migrant background. Only caregivers with a contract for at least half-time work and involvement in the daily care of people with dementia in all stages of the disease were eligible to participate.

#### Evaluation of the IRR of the QUALIDEM

IRR data were collected in a convenience sample from nine nursing homes. The sample size calculation for the IRR evaluation was based on intra-class correlation (ICC) values for the QUALIDEM subscales in an earlier work [19], ratings of four independent proxy raters (professional caregivers) and a width of 0.20 for a 95 % confidence interval (CI). The calculated IRR sample varied depending on the QUALIDEM subscale being tested. Seventy-nine to 115 residents with mild to severe dementia were needed for the 37-item version, and 65 to 114 residents with very severe dementia were needed for the 18-item version [21]. The inclusion criteria for residents with dementia consisted of a recorded dementia diagnosis, a Functional Assessment Staging (FAST) [22] value ≥ 2 and residence in the nursing home for at least two weeks. The qualification levels of the proxy raters (registered nurses and nursing assistants) depended on the organizational conditions and staffing levels at the time of data collection. The following inclusion criteria for caregivers were used: a close relationship with the assessed resident and a contract for at least half-time work. Both of these criteria were assessed based on information provided by the caregivers. To facilitate the collection of up-to-date information and to ensure that the clients were observed in the same time period, the nurses had to have worked most of the days in the 2 weeks prior to the data collection. Based on these criteria, the caregivers were recruited by the nursing home’s management team for the cognitive interviews and for IRR evaluation.

### Procedures

#### Development of the QUALIDEM user guide

For the cognitive interviews, we conducted one focus group interview with six caregivers from two nursing homes. These caregivers were experienced in the regular application of the QUALIDEM over a period of ≥ 2 years prior to the data collection. We also conducted individual interviews with ten caregivers from four nursing homes who were not familiar with the QUALIDEM prior to the interview. The interviews were electronically recorded. At the beginning of the interviews, each caregiver assessed the Qol of a resident with dementia whom he/she knew well. After the instrument was used, the researcher reviewed the responses for missing data, irregularities (e.g., items marked twice) or additional handwritten comments. When irregularities were found, the caregivers were asked about the reasons for these inconsistencies. Each participant’s understanding of each item was assessed using verbal probes. These cognitive probes prompted caregivers to reflect on the meanings of items or the reasons for and backgrounds for their ratings [23]. During these interviews, the caregivers provided many examples for each item in the user guide. The same approach was used for the individual and focus-group-based interviews with experienced and inexperienced caregivers.

#### Evaluation of the IRR of the QUALIDEM

Proxy ratings from caregivers referring to the week prior to the QUALIDEM ratings were used to evaluate the IRR. The Qol of each participating resident with dementia were assessed by four different caregivers. Each caregiver was blinded to the ratings of the other proxy raters. To ensure standardized data collection and blinding of the proxy raters, QUALIDEM application was introduced by the first author (MND). At the start of the Qol ratings, each caregiver received an explanation of the meaning of each QUALIDEM item with examples and corresponding response options. This explanation was based on the QUALIDEM user guide developed in the first step. In addition, the caregivers received a version of the QUALIDEM user guide. Thus, while providing Qol ratings, the caregivers could clarify the interpretation of an item by reading the definition and examples for each item in the user guide.

### Measurements

#### Development of the QUALIDEM user guide

The cognitive interviews (individual interviews and a focus group-based interview) were based on the flexible application of six cognitive probes recommended by Willis [23]. In accordance with Hoben et al. [24], we developed an example question for each probe and each QUALIDEM item prior to the interviews. For item 1 on the QUALIDEM, ‘is cheerful’, the following example questions were used:

- ▪ What does ‘is cheerful’ mean to you (comprehension/interpretation probe)?
- ▪ Can you repeat the question in your own words (paraphrasing probe)?
- ▪ How sure are you that the person with dementia was cheerful in the last week (confidence judgment probe)?
- ▪ How do you remember the person with dementia as cheerful in the last week (recall probe)?
- ▪ Why do you think that the person with dementia was cheerful (specific probe)?
- ▪ Was it easy or hard to answer?
- ▪ What did you think when answering the question (general probe)?

These example questions were used as a flexible interview guide. The interviewer chose the type of probe and asked additional questions based on the item and the interview situation. Moreover, with respect to the QUALIDEM items, the applicability of the QUALIDEM response options and the underlying observation period of the ratings were questioned as part of the cognitive interviews.

#### Evaluation of the IRR of the QUALIDEM

The QUALIDEM consists of two consecutive versions that cover nine Qol domains (‘care relationship’, ‘positive affect’, ‘negative affect’, ‘restless tense behavior’, ‘positive self-image’, ‘social relations’, ‘social isolation’, ‘feeling at home’, and ‘having something to do’) for people with mild to severe dementia and six domains (excluding ‘positive self-image’, ‘feeling at home’, and ‘having something to do’) for people with very severe dementia. In this IRR study, we also tested three additional QUALIDEM items that were not scalable during the development of the instrument but were recommended for further research [14]. The stage of dementia severity was assessed using the FAST, which is used to assess seven severity stages (1 = free of cognitive impairment, 2 – 6 = mild to severe dementia, 7 = very severe dementia) [22]. Functional ability was assessed using the Physical Self-Maintenance Scale (PSMS), which results in a score between 6 and 30, with higher scores indicating lower functional ability [25]. Residents’ care dependency was assessed using the levels defined by the German statutory long-term care insurance system (range: 1 = low to 3 = high). Socio-demographic data were collected for residents and all professional caregivers who rated the QUALIDEM items.

### Data analysis

#### Development of the QUALIDEM user guide

The recorded interviews were summarized using content analysis [26]. The participants’ responses to the cognitive probes were summarized as statements about the participants’ understandings of the QUALIDEM items (e.g., misinterpretations, definitions or parts of definitions, examples to describe the item meaning) and the applicability of the QUALIDEM response options. The results were the basis for the formulation of the item definitions and the development of typical item examples. If warranted, reformulation of the items and changes to the QUALIDEM response options and the underlying observation periods were also considered. All of the possible changes of the instrument were considered to increase the clarity and reliability of the item ratings and the precision of the item definitions and examples. The definitions and examples for all of the QUALIDEM items and the reformulation of items were discussed in a 1-day workshop with the first author of the original QUALIDEM to develop the final definitions and examples.

#### Evaluation of the IRR of the QUALIDEM

The sample characteristics are presented using descriptive statistics. Item distributions, means and standard deviations (SDs) were calculated. The internal consistency of the QUALIDEM subscales was analyzed using Cronbach’s alpha based on the medians of four independent observations for each case and item. The IRR analysis was based on the procedures of two earlier IRR studies that compared the IRR results for items [19] and subscales [14, 19]. To determine the IRR for each item, the mean overall proportion of agreement (p_o_) was calculated. This means, that the four independent observations by different caregivers resulted in an analysis which was based on six different rater pairs to compute the ratio of exact agreement between raters to the total number of ratings (p_o_). Because the p_o_ ignores the possibility that agreement could occur only by chance and instead considers only crude agreement, we computed the multi-rater k statistics for ordinal data (k, i.e., Conger’s kappa) [27]. The two paradoxical properties of k statistics were also considered during the interpretation of the results [28]. The IRRs of the QUALIDEM subscales were evaluated using ICCs based on a two-way random-effects model for absolute agreement. Based on the recommendation by Terwee et al. [29], we targeted kappa and ICC values ≥ 0.7. Furthermore, our interpretation of kappa values was based on the following recommendation by Landis and Koch [30]: 0.00 – 0.20, slight; 0.21 – 0.40, fair; 0.41 – 0.60, moderate; 0.61 – 0.80, substantial; and 0.81 – 1.00, nearly perfect. To analyze the level of uncertainty, 95 % CIs for ICCs and k values were examined. The CIs for k values were based on 10,000 bootstrapped samples [31]. We drew 10,000 resamples as a replacement with the same size as the original sample. The k statistic was then calculated for each resample. The bootstrap 95 % CI was determined using the percentile method [32], which included using the 0.025 and 0.975 percentile levels of the estimated kappa distributions as interval limits.

The SPSS Statistics version 21 and R [33–36] software packages were used for the statistical analyses.

## Results

### Development of the QUALIDEM user guide

The sample for the cognitive interviews consists of 16 caregivers from six nursing homes. The sample characteristics are described in Table 1.

**Table 1:** Characteristics of the sample

Proxy raters	Cognitive interviews (*n* = 16)	IRR evaluation (*n* = 40)
Age in years, mean (±SD)	45.0 (±11.2)	41.7 (±12.0)
Female, *n* (%)	11 (69)	34 (85)
Registered nurses, *n* (%)	10 (63)	24 (60)
Work experience in years, mean (±SD)	14.3 (±7.5)	11.8 (±7.3)
Hours worked per week, mean (±SD)	35.0 (±4.7)	32.7 (±5.5)
Caregivers with migrant backgrounds, *n* (%)	4 (25)	---
People with dementia	Mild to severe dementia (*n* = 55)	Very severe dementia (*n* = 36)
Age in years, mean (±SD)	84.7 (±8.3)	84.1 (±9.4)
Female, *n* (%)	43 (78)	33 (92)
PSMS, mean (± SD)	19.8 (±4.6)	24.9 (±2.3)
Care dependency levels^a^, *n* (%)		
None	0 (0)	0 (0)
1	7 (13)	0 (0)
2	29 (53)	5 (14)
3	19 (34)	31 (86)
FAST score, *n* (%)		
2	1 (2)	
3	1 (2)	
4	3 (5)	
5	0 (0)	
6	50 (91)	
Length of stay in the nursing home in months, mean (±SD)	26.6 (±21.6)	40.1 (±23.2)
Missing values, QUALIDEM^b^	0/8800	7/3024

^a^As determined by expert raters of the medical service of the statutory long-term care insurance system

^b^Each QUALIDEM item was rated four times for each participant (FAST 2 – 6 = 37 + 3 items, FAST 7 = 18 + 3 items)

The cognitive interviews revealed that the meaning of three items had been misinterpreted. The previous German translations of the terms ‘restless’ (items 2, 19) and ‘friendly terms’ (item 29) resulted in misinterpretations by caregivers. Based on the previous translations, interview participants rated under item 29 a possible friendship between one or more residents and not the originally intended item meaning ‘Is on friendly terms with one or more residents’. Moreover, caregivers with a migration background understood item 2 ‘Makes restless movements’ to have the opposite meaning, i.e., ‘Makes calm movements’. Therefore, the wording of these items was altered in consultation with the first author of the original QUALIDEM and two translators (two nursing scientists, one with German as a first language and excellent Dutch language skills and the other with Dutch as a first language and excellent German language skills).

Several items led to ambiguities throughout the Qol ratings. For instance, for the interview participants, the extent to which the calling of a resident had to be targeted or untargeted was unclear (item 32). Another example was an ambiguity related to item 1. Here, it was unclear if cheerfulness referred to a positive mood expressed over a long time period or to a result of a short-term nursing intervention. Based on the results of the cognitive interviews, the item definitions and examples [see Additional file 1] were developed as described above.

The different time frames of the recommended observation period of the original QUALIDEM version (2 weeks) and the item response options (1 week) were confusing for proxy-rating caregivers who lacked experience in the application of the QUALIDEM and promoted uncertainty in the ratings they provided.

Moreover, caregivers with experience in the application of the QUALIDEM criticized the four response options of the original QUALIDEM (never, seldom, sometimes, and often) as insufficiently differentiated for an accurate assessment.

As result of the cognitive interviews, we changed the response options from a four-option scale to a seven-option scale (ranging from never to very frequently) to enhance the sensitivity of the QUALIDEM’s ratings. Table 2 presents the new seven response options and their definitions along with the original four response options and their definitions. Furthermore, we reduced the underlying observation period for the ratings to 1 week.

**Table 2:** Comparison of QUALIDEM response options

Seven response options		Original four response options [14]	
Response options	Definition	Response options	Definition
Never	Never	Never	Never
Very seldom	No more than once a week	Seldom	No more than once a week
Seldom	Two to three times a week	Sometimes	A few times per week
Sometimes	Four to five times a week		
Often	Almost daily	Often	Almost daily
Frequently	Daily		
Very frequently	Several times a day		

### Evaluation of the IRR of the QUALIDEM

To evaluate the IRR, the sample was divided into one sample for people with mild to severe dementia (*n* = 55) and one sample for people with very severe dementia (*n* = 36). As described above the Qol of each participant was rated by four different caregivers. These proxy ratings were done by all together 40 caregivers who were included in the IRR evaluation based on the predefined inclusion criteria. Table 1 presents a description of the characteristics of the proxy raters and people with dementia.

#### Item distribution and internal consistency

The descriptive analysis of the QUALIDEM items revealed a skewed distribution (Table 3). The response option ‘never’ was used most frequently; the distribution of the other response options was balanced, and no tendency was observed for a middle response option. Based on the mean values, one item showed a floor effect (item 38, people with mild to severe dementia) and 12 items (item 12, 13, 16, 23, 27, 28, 31, 32, 33, 35, 37, 39) and six items (item 12, 16, 20, 23, 25, 31) in the two respective versions showed ceiling effects.

**Table 3:** Item distribution and internal consistency per item based on four observations for each case – German version of the QUALIDEM

			FAST 2 – 6 (*n* = 55)										FAST 7 (*n* = 36)									
			Never	Very seldom	Seldom	Sometimes	Often	Frequently	Very frequently	Mean	SD	Alpha^c^	Never	Very seldom	Seldom	Sometimes	Often	Frequently	Very frequently	Mean	SD	Alpha^c^
**A.**	**Care relationship**											**0.9**										**0.8**
	4	Rejects help from nursing assistants^a^	79	48	29	23	12	13	16	4.3	1.9											
	7	Is angry^a^	31	46	61	36	14	12	20	3.7	1.7		59	39	9	13	12	4	8	4.5	1.8	
	14	Has conflicts with nursing assistants^a^	93	65	21	16	5	9	11	4.7	1.7		78	19	12	12	10	4	9	4.7	1.9	
	17	Accuses others^a^	90	43	29	23	14	17	4	4.5	1.7											
	24	Appreciates help that he or she receives	5	4	18	35	31	55	72	4.4	1.5											
	31	Accepts help	0	4	1	14	24	50	127	5.3^b^	1.1		1	1	4	4	11	34	89	5.3^b^	1.1	
	33	Criticizes the daily routine^a^	147	30	14	5	13	8	3	5.2^b^	1.5											
**B.**	**Positive Affect**											**0.9**										**0.8**
	1	Is cheerful	19	20	24	38	49	40	30	3.5	1.8											
	5	Radiates satisfaction	4	10	18	21	65	63	39	4.2	1.5		10	10	13	5	32	49	25	4.0	1.8	
	8	Is capable of enjoying things in daily life	2	0	13	25	53	76	51	4.5	1.2		9	7	6	21	31	49	21	4.0	1.6	
	10	Is in good mood	10	19	19	40	55	51	26	3.7	1.6											
	21	Has a smile around the mouth	9	17	28	31	38	46	51	3.9	1.8		12	9	24	17	20	27	35	3.7	1.9	
	40	Mood can be influenced in positive sense	8	13	15	22	44	71	47	4.2	1.6		10	15	9	8	14	43	45	4.2	2.0	
**C.**	**Negative Affect**											**0.7**										**0.4**
	6	Makes an anxious impression^a^	66	57	35	33	20	8	1	4.4	1.5		24	15	17	29	21	17	21	3.0	2.0	
	11	Is sad^a^	69	42	31	32	23	16	7	4.1	1.8											
	23	Cries^a^	143	24	17	24	12	0	0	5.2^b^	1.3		113	16	0	10	2	1	2	5.5^b^	1.2	
**D.**	**Restless tense behavior**											**0.8**										**0.7**
	2	Makes restless movements^a^	89	29	20	17	14	14	37	3.9	2.3		38	15	11	8	14	22	36	2.9	2.4	
	19	Is restless^a^	74	52	19	18	9	16	32	4.0	2.2		28	23	21	3	12	17	40	2.9	2.4	
	22	Has tense body language^a^	93	35	33	31	17	4	7	4.5	1.6		28	12	20	13	8	31	32	2.7	2.3	
**E.**	**Positive self-image**											**0.7**										
	27	Indicates he or she would like more help^a^	165	29	10	9	5	2	0	5.5^b^	1.0											
	35	Indicates not being able to do anything^a^	129	38	23	13	12	4	1	5.1^b^	1.4											
	37	Indicates feeling worthless^a^	134	20	11	28	16	7	4	4.9^b^	1.7											
**F.**	**Social relations**											**0.8**										**0.4**
	3	Has contact with other residents	16	12	15	8	30	47	92	4.4	1.9		57	22	14	3	0	26	22	2.2	2.4	
	12	Responds positively when approached	1	2	7	26	30	61	93	4.9^b^	1.3		4	3	5	11	13	45	63	4.9^b^	1.5	
	18	Takes care for other residents	107	27	20	17	19	19	11	1.6	2.0											
	25	Cuts himself/herself off from environment	99	31	29	27	10	13	11	4.5	1.8		116	10	2	2	4	4	6	5.4^b^	1.6	
	29	Is on friendly terms with one or more residents	56	17	15	25	19	29	59	3.2	2.4											
	34	Feels at ease in the company of others	13	27	14	12	41	64	49	4.0	1.9											
**G.**	**Social isolation**											**0.5**										**0.1**
	16	Is rejected by other residents^a^	124	45	5	21	4	11	10	4.9^b^	1.8		104	19	7	5	4	2	3	5.4^b^	1.3	
	20	Openly rejects contact with others^a^	68	46	32	18	27	23	6	4.1	1.8		90	25	11	12	1	3	2	5.2^b^	1.3	
	32	Calls out^b^	167	13	5	4	4	6	21	5.1^b^	2.0		97	7	5	1	1	3	30	4.5	2.5	
**H.**	**Feeling at home**											**0.4**										
	13	Indicates that he or she is bored^a^	148	43	23	5	1	0	0	5.5^b^	0.8											
	28	Indicates feeling locked up^a^	181	16	13	5	5	0	0	5.7^b^	0.9											
	36	Feels at home on the ward	61	21	19	17	23	53	26	2.8	2.3											
	39	Wants to get off the ward^a^	172	20	9	6	7	2	4	5.5^b^	1.3											
**I.**	**Having something to do**											**0.3**										
	26	Finds things to do without help from others	77	13	11	21	16	38	44	2.8	2.4											
	38	Enjoys helping with chores on the ward	118	43	18	17	9	13	2	1.1^b^	1.6											
	**Remaining items to be used in future research**																					
	9	Does not want to eat^a^	105	48	14	18	16	17	2	4.7	1.7		53	25	31	13	5	5	11	4.3	1.8	
	15	Enjoys meals	10	15	27	18	42	54	54	4.0	1.8		17	12	10	17	10	41	35	3.8	2.1	
	30	Likes to lie down (in bed)^a^	81	30	17	24	22	20	26	3.8	2.2		69	19	7	7	21	10	7	4.4	2.0	
Frequencies of the response options in total, n			3063	1114	782	823	870	1052	1096				1017	323	238	214	246	437	542			
Frequencies of the response options in total, %			35	13	9	9	10	12	12				34	11	8	7	8	14	18			

^a^Contraindicative items

^b^Items with floor effects (mean < 1.2) or ceiling effects (mean > 4.8)

^c^Calculation of Cronbach’s alpha based on the median of four observations for each case and item

Cronbach’s alpha values ranged from 0.3 to 0.9 (37-item version) and from 0.1 to 0.8 (18-item version).

#### Inter-rater reliability

Nearly all of the QUALIDEM subscales had strong IRR based on their ICC values for people with either mild to severe or very severe dementia. A moderate IRR was identified only for the ‘negative affect’ subscale for people with very severe dementia. These positive results were also confirmed after excluding items with floor or ceiling effects (Table 4).

**Table 4:** Inter-rater reliability results for the German version of the QUALIDEM

Inter-rater reliability: Subscales and items			FAST 2 – 6 (*n* = 55)				FAST 7 (*n* = 36)			
			p_o_^a^	ICC^b^	k^c^	95 % CI	p_o_^a^	ICC^b^	k^c^	95 % CI
**A.**	**Care relationship**			**0.92 (0.91)** ^**e**^		**0.89 – 0.95 (0.87 – 0.94)** ^**e**^		**0.79 (0.78)** ^**e**^		**0.68 – 0.87 (0.67 – 0.87)** ^**e**^
	4	Rejects help from nursing assistants	0.61		0.50	0.40 – 0.59				
	7	Is angry	0.60		0.52	0.42 – 0.60	0.63		0.49	0.35 – 0.62
	14	Has conflicts with nursing assistants	0.56		0.38	0.29 – 0.47	0.66		0.49	0.34 – 0.63
	17	Accuses others	0.63		0.52	0.42 – 0.61				
	24	Appreciates help that he or she receives	0.55		0.42	0.31 – 0.52				
	31	Accepts help	0.62^d^		0.38^d^	0.23 – 0.51	0.67^d^		0.41^d^	0.27 – 0.53
	33	Criticizes the daily routine	0.67^d^		0.38^d^	0.20 – 0.53				
**B.**	**Positive affect**			**0.91**		**0.87 – 0.94**		**0.82**		**0.73 – 0.90**
	1	Is cheerful	0.49		0.39	0.31 – 0.46				
	5	Radiates satisfaction	0.52		0.38	0.29 – 0.47	0.44		0.29	0.19 – 0.38
	8	Is capable of enjoying things in daily life	0.55		0.40	0.29 – 0.50	0.57		0.46	0.32 – 0.59
	10	Is in good mood	0.54		0.44	0.36 – 0.52				
	21	Has a smile around the mouth	0.51		0.41	0.33 – 0.48	0.61		0.53	0.39 – 0.66
	40	Mood can be influenced in positive sense	0.48		0.34	0.24 – 0.43	0.46		0.32	0.19 – 0.43
**C.**	**Negative affect**			**0.91 (0.89)** ^**e**^		**0.87 – 0.94 (0.84 – 0.93)** ^**e**^		**0.64**		**0.49 – 0.77**
	6	Makes an anxious impression	0.61		0.50	0.39 – 0.59	0.56		0.48	0.37 – 0.58
	11	Is sad	0.57		0.47	0.37 – 0.55				
	23	Cries	0.77^d^		0.58^d^	0.46 – 0.69	0.83^d^		0.54^d^	0.28 – 0.75
**D.**	**Restless tense behavior**			**0.95**		**0.92 – 0.97**		**0.91**		**0.85 – 0.95**
	2	Makes restless movements	0.64		0.53	0.43 – 0.62	0.65		0.58	0.47 – 0.67
	19	Is restless	0.55		0.43	0.34 – 0.52	0.62		0.54	0.41 – 0.65
	22	Has tense body language	0.68		0.58	0.45 – 0.68	0.53		0.44	0.31 – 0.55
**E.**	**Positive self-image**			**0.92**		**0.88 – 0.95**				
	27	Indicates he or she would like more help	0.77^d^		0.45^d^	0.27 – 0.59				
	35	Indicates not being able to do anything	0.66^d^		0.45^d^	0.32 – 0.57				
	37	Indicates feeling worthless	0.74^d^		0.57^d^	0.48 – 0.65				
**F.**	**Social relations**			**0.93 (0.93)** ^**e**^		**0.89 – 0.95 (0.90 – 0.95)** ^**e**^		**0.83**		**0.74 – 0.90**
	3	Has contact with other residents	0.60		0.46	0.34 – 0.57	0.59		0.47	0.35 – 0.56
	12	Responds positively when approached	0.55^d^		0.37^d^	0.26 – 0.46	0.59^d^		0.41^d^	0.25 – 0.56
	18	Takes care for other residents	0.69		0.57	0.47 – 0.66				
	25	Cuts himself/herself off from environment	0.64		0.51	0.41 – 0.60	0.79^d^		0.40^d^	0.14 – 0.59
	29	Is on friendly terms with one or more residents	0.68		0.60	0.51 – 0.69				
	34	Feels at ease in the company of others	0.44		0.31	0.22 – 0.39				
**G.**	**Social isolation**			**0.95**		**0.92 – 0.97**		**0.84**		**0.75 – 0.90**
	16	Is rejected by other residents	0.68^d^		0.49^d^	0.36 – 0.60	0.75^d^		0.44^d^	0.20 – 0.63
	20	Openly rejects contact with others	0.55		0.45	0.36 – 0.52	0.64^d^		0.37^d^	0.18 – 0.52
	32	Calls out	0.85^d^		0.62^d^	0.44 – 0.76	0.82		0.65	0.46 – 0.80
**H.**	**Feeling at home**			**0.91**		**0.87 – 0.94**				
	13	Indicates that he or she is bored	0.70^d^		0.40^d^	0.26 – 0.52				
	28	Indicates feeling locked up	0.85^d^		0.52^d^	0.31 – 0.67				
	36	Feels at home on the ward	0.62		0.54	0.44 – 0.63				
	39	Wants to get off the ward	0.82^d^		0.52^d^	0.36 – 0.65				
**I.**	**Having something to do**			**0.96**		**0.94 – 0.97**				
	26	Finds things to do without help from others	0.69		0.61	0.51 – 0.70				
	38	Enjoys helping with chores on the ward	0.70^d^		0.55^d^	0.43 – 0.66				
	**Remaining items to be used in future research**									
	9	Does not want to eat	0.63		0.47	0.36 – 0.58	0.63		0.52	0.37 – 0.65
	15	Enjoys meals	0.58		0.49	0.39 – 0.58	0.54		0.43	0.29 – 0.55
	30	Likes to lie down (in bed)	0.55		0.44	0.35 – 0.53	0.59		0.42	0.27 – 0.56

^a^Overall proportion of agreement (the ratio of exact agreement between raters to the total number of ratings)

^b^ *ICC* intra-class correlation coefficient

^c^Kappa values

^d^Items with floor effects (mean < 1.2) or ceiling effects (mean > 4.8), see Table 3

^e^ICC values and corresponding 95 % CIs when excluding items with floor or ceiling effects

The IRR results for each item based on p_o_ ranged from 0.44 (item 34) to 0.85 (items 28 and 32) for people with mild to severe dementia and from 0.44 (item 5) to 0.83 (item 23) for those with very severe dementia. The k values ranged from 0.31 (item 34) to 0.62 (item 32) for the 37-item version and from 0.32 (item 40) to 0.65 (item 32) for the 18-item version. To ensure comparability with previous studies, we reanalyzed the k values for each item based on the original four response options. Based on the original four response options, the k values ranged from 0.43 (item 33) to 0.80 (item 26) for the 37-item version and from 0.42 (item 20) to 0.80 (item 26) for the 18-item version [see Additional file 2].

## Discussion

### Development of the QUALIDEM user guide

The results of previous studies [14, 19] suggest that some of the QUALIDEM items are not well understood. Therefore, a comprehensive user guide for the German version of the QUALIDEM was developed for observation-based Qol ratings. After the introduction of the user guide, the nine subscales of the QUALIDEM version for people with mild to severe dementia and the six subscales for those with very severe dementia showed strong IRR (ICC: 0.79–0.96), with the exception of the ‘negative affect’ subscale (people with very severe dementia), which was found to have a moderate level of IRR (ICC: 0.64). Compared to previous results [14, 19] based on ICC values for absolute agreement, our results demonstrate a significant improvement in the IRR of the German QUALIDEM version. This study shows that assessment instruments should be used only when applicants understand the underlying meaning (i.e., the theoretical basis) of the instrument’s items.

### Evaluation of the IRR of the QUALIDEM

Ten items for people with mild to severe dementia showed fair IRR (k = 0.21 – 0.40: 1, 5, 8, 12 – 14, 31, 33, 34, 40), 28 items were found to have moderate IRR (k = 0.41 – 0.60: 2 – 4, 6, 7, 9 – 11, 15 – 25, 27 – 30, 35 – 39) and two items were found to have substantial IRR (k = 0.61 – 0.80: 26, 32). For people with very severe dementia, the IRR was fair for four items (items 5, 20, 25, 40), moderate for 16 items (items 2, 3, 6 – 9, 12, 14 – 16, 19, 21 – 23, 30, 31), and substantial for one item (item 32). In summary, the IRR results for each QUALIDEM item showed an average improvement of approximately 0.1 for each k value when compared to a previous reliability study [19]. Notably, these results are based on seven response options, whereas the IRR results of previous studies were based on four response options. A reanalysis of k values for each item based on the original four response options results in greater IRR improvements for each item. Thus, analyzing QUALIDEM values on the subscale level using seven response options appears to be appropriate because of the strong or moderate IRR for all subscales and the extended discriminatory power of the seven response options.

The IRR results were sufficient in comparison to other dementia-specific Qol instruments. Strong IRR results were found for the Alzheimer Disease-Related Quality of Life (ADRQL; ICC: 0.90 – 1.00) instrument, the Quality of Life – Alzheimer’s Disease Scale for Nursing Homes (QoL-AD NH; ICC: 0.99) and the Affect and Activity Indicators of Quality of Life (AAIQOL; ICC 0.66 – 0.78) instrument in a US nursing home setting [37]. As with our IRR results, the IRR results for other dementia-specific Qol instruments vary also depending on the culture-specific version and usage. Compared with the above-mentioned results, Menzi-Kuhn [38] observed weak IRR for the US version of the instrument in a study of the Swiss version of the ADRQL. For the Quality of Life in Late-stage Dementia Scale (QUALID), the IRR results included ICC values of 0.83 for the US version [39], 0.74 for the Spanish version [40] and 0.69 for the Swedish version [41].

All of these instruments differ depending on the Qol domains assessed and their feasibility [7]. Methodological limitations such as small sample sizes (≤ 25) limit the interpretation of these IRR results [15]. The majority of these instruments are accompanied by a user guide that includes general recommendations for application of the instrument and may provide examples for the interpretation of items. Through our study, a user guide with definitions and examples for each item is now available for the German version of the QUALIDEM. The application of the user guide increased the time required for the first Qol ratings until caregivers have to memorize all of the item definitions. The improvement of the IRR may be considered justification for using the guide. Moreover, the application of the user guide now allows QUALIDEM ratings to be provided by single caregivers in research. This will lead to a reduction in the effort required to obtain acceptable IRR results in research. Before the present study, a collaborative QUALIDEM rating based on the ratings of two or more caregivers was recommended [14, 19]. Furthermore, the rating of Qol for people with dementia is a complex and costly process. Researchers must consider the challenges inherent in rating before determining the Qol outcome and adapt their methodological approaches accordingly.

Beyond the sufficient IRR results, the descriptive results provide information relevant to the further development of the QUALIDEM. The floor and ceiling effects for 13 items (12, 13, 16, 23, 27, 28, 31, 32, 33, 35, 37, 38, 39) for people with mild to severe dementia and six items (12, 16, 20, 23, 25, 31) for people with very severe dementia indicate that these items are less informative when based on seven response options. In particular, the exclusion and reformulation of items 12 and 31 must be considered in the subsequent development of the instrument because ceiling effects for these items were also found in the first pilot study of the QUALIDEM [42]. The descriptive results for the items 13, 16, 20, 23, 25, 27, 28, 32, 33, 35, 37, 38 and 39 require confirmation in further studies. Moreover, secondary data analysis of existing data sets at the item level is recommended, as item-level analyses are lacking [14, 16 – 18, 20, 43]. The weak internal consistency results for ‘social isolation’, ‘feeling at home’, and ‘having something to do’ are consistent with previous results [20] and indicate the need for the further development and investigation of these subscales for the German QUALIDEM. The internal consistency results for the Dutch versions of these subscales are heterogeneous [14, 18]. The rejection of the ‘social isolation’ subscale should be considered because of the less informative items on this subscale and the content overlap with the ‘social relations’ subscale.

### Limitations

The strength of this IRR study is the high number of QUALIDEM ratings based on four proxy raters for each resident. The preplanned sample size was not fulfilled (FAST 2 – 6: 70 %, FAST 7: 55 %) because of the time-consuming nature of the data collection in the participating nursing homes (four Qol ratings from different caregivers for each resident). However, the narrow CIs indicate that the sample was sufficient. The maximum ICC CI lengths for people with mild to severe dementia and those with very severe dementia were 0.07 and 0.28, respectively.

Given the relatively small number of residents with dementia included in the study, caution must be exercised in interpreting the item distributions. However, the socio-demographic characteristics of the included residents are comparable to those of other studies in this field [19].

## Conclusions

The application of the user guide developed for the German version of the QUALIDEM resulted in sufficient IRR results for the QUALIDEM subscales. Only the ‘negative affect’ subscale for people with very severe dementia was found to have a moderate IRR. The IRR results for the QUALIDEM items were found to be fair to substantial for both QUALIDEM versions. Thus, the subscales of the German version of the QUALIDEM can be assumed to have sufficient IRR if the proxy rating is based on the user guide recommendations. These results indicate that the application of the user guide allows QUALIDEM ratings by single caregivers to be acceptable in research.

However, the item distribution and internal consistency results highlight the need for further development and investigation of the items for the ‘social isolation’, ‘feeling at home’ and ‘having something to do’ subscales, particularly for the German QUALIDEM.

Through collaboration between the authors of the original Dutch version of the QUALIDEM and the authors of this IRR study, a linguistically validated English language version of the QUALIDEM user guide will soon be available.

## Abbreviations

CI, confidence interval; FAST, functional assessment staging; ICC, intra-class correlation coefficient; IRR, inter-rater reliability; p_o_, proportion of overall agreement; PSMS, physical self-maintenance scale; Qol, quality of life; SD, standard deviation; k, kappa statistics

## Additional files

Additional file 1: Definition and examples of the German QUALIDEM items. (DOCX 63 kb) Additional file 2: Inter-rater reliability results for the German version of the QUALIDEM – reanalysis based on 4 response options. (DOCX 44 kb)
