# Supplementary material for: Item distribution, internal consistency and inter-rater reliability of the German version of the QUALIDEM for people with mild to severe and very severe dementia
Source: BMC Geriatr. 2016 Jun 18;16:126. doi: 10.1186/s12877-016-0296-0 (PMC4912742; doi:10.1186/s12877-016-0296-0)
Supplement: Additional file 1: — Definition and examples of the German QUALIDEM items. (DOCX 63 kb) [file 12877_2016_296_MOESM1_ESM.docx]

## Additional file 2: Inter-rater reliability results for the German version of the QUALIDEM – reanalysis based on 4 response options

| **Inter-rater reliability: Subscales and items** | | | **FAST 2 – 6 (n = 55)** | | | | **FAST 7 (n = 36)** | | | |
| --- | --- | --- | --- | --- | --- | --- | --- | --- | --- | --- |
|  |  | | **p_o_^a^** | **ICC^b^** | **κ^c^** | **95% CI** | **p_o_^a^** | **ICC^b^** | **κ^c^** | **95% CI** |
| **A.** | **Care relationship** | |  | **0.90** |  | **0.86 – 0.94** |  | **0.82** |  | **0.73 – 0.89** |
|  | 4 | Rejects help from nursing assistants | 0.73 |  | 0.63 | 0.51 – 0.73 |  |  |  |  |
|  | 7 | Is angry | 0.73 |  | 0.62 | 0.51 – 0.72 | 0.66 |  | 0.52 | 0.37 – 0.66 |
|  | 14 | Has conflicts with nursing assistants | 0.63 |  | 0.48 | 0.35 – 0.58 | 0.73 |  | 0.58 | 0.41 – 0.72 |
|  | 17 | Accuses others | 0.73 |  | 0.63 | 0.51 – 0.74 |  |  |  |  |
|  | 24 | Appreciates help that he or she receives | 0.87 |  | 0.72 | 0.56 – 0.85 |  |  |  |  |
|  | 31 | Accepts help | 0.95 |  | 0.72 | 0.27 – 0.94 | 0.93 |  | 0.47 | 0.00 – 0.86 |
|  | 33 | Criticizes the daily routine | 0.70 |  | 0.43 | 0.23 – 0.58 |  |  |  |  |
| **B.** | **Positive affect** | |  | **0.90** |  | **0.86 – 0.94** |  | **0.83** |  | **0.74 – 0.90** |
|  | 1 | Is cheerful | 0.76 |  | 0.61 | 0.51 – 0.71 |  |  |  |  |
|  | 5 | Radiates satisfaction | 0.86 |  | 0.64 | 0.47 – 0.78 | 0.81 |  | 0.57 | 0.36 – 0.75 |
|  | 8 | Is capable of enjoying things in daily life | 0.92 |  | 0.74 | 0.57 – 0.88 | 0.80 |  | 0.58 | 0.39 – 0.74 |
|  | 10 | Is in good mood | 0.79 |  | 0.63 | 0.52 – 0.73 |  |  |  |  |
|  | 21 | Has a smile around the mouth | 0.81 |  | 0.65 | 0.53 – 0.77 | 0.86 |  | 0.76 | 0.61 – 0.90 |
|  | 40 | Mood can be influenced in positive sense | 0.86 |  | 0.69 | 0.58 – 0.81 | 0.85 |  | 0.68 | 0.51 – 0.83 |
| **C.** | **Negative affect** | |  | **0.89** |  | **0.84 – 0.93** |  | **0.67** |  | **0.53 – 0.80** |
|  | 6 | Makes an anxious impression | 0.66 |  | 0.54 | 0.43 – 0.65 | 0.78 |  | 0.69 | 0.55 – 0.81 |
|  | 11 | Is sad | 0.69 |  | 0.58 | 0.47 – 0.68 |  |  |  |  |
|  | 23 | Cries | 0.80 |  | 0.62 | 0.49 – 0.74 | 0.83 |  | 0.55 | 0.29 – 0.76 |
| **D.** | **Restless tense behavior** | |  | **0.92** |  | **0.86 – 0.95** |  | **0.89** |  | **0.83 – 0.94** |
|  | 2 | Makes restless movements | 0.78 |  | 0.70 | 0.58 – 0.80 | 0.82 |  | 0.74 | 0.62 – 0.84 |
|  | 19 | Is restless | 0.70 |  | 0.60 | 0.48 – 0.70 | 0.76 |  | 0.65 | 0.51 – 0.78 |
|  | 22 | Has tense body language | 0.74 |  | 0.63 | 0.50 – 0.74 | 0.75 |  | 0.63 | 0.49 – 0.75 |
| **E.** | **Positive self-image** | |  | **0.90** |  | **0.86 – 0.94** |  |  |  |  |
|  | 27 | Indicates he or she would like more help | 0.79 |  | 0.50 | 0.31 – 0.66 |  |  |  |  |
|  | 35 | Indicates not being able to do anything | 0.72 |  | 0.54 | 0.38 – 0.67 |  |  |  |  |
|  | 37 | Indicates feeling worthless | 0.82 |  | 0.70 | 0.58 – 0.80 |  |  |  |  |
| **F.** | **Social relations** | |  | **0.93** |  | **0.90 – 0.96** |  | **0.84** |  | **0.75 – 0.90** |
|  | 3 | Has contact with other residents | 0.90 |  | 0.75 | 0.60 – 0.88 | 0.76 |  | 0.67 | 0.52 – 0.80 |
|  | 12 | Responds positively when approached | 0.89 |  | 0.63 | 0.45 – 0.79 | 0.90 |  | 0.67 | 0.42 – 0.85 |
|  | 18 | Takes care for other residents | 0.80 |  | 0.72 | 0.60 – 0.82 |  |  |  |  |
|  | 25 | Cuts himself/herself off from environment | 0.73 |  | 0.61 | 0.49 – 0.72 | 0.81 |  | 0.45 | 0.17 – 0.66 |
|  | 29 | Is on friendly terms with one or more residents | 0.84 |  | 0.76 | 0.65 – 0.86 |  |  |  |  |
|  | 34 | Feels at ease in the company of others | 0.80 |  | 0.59 | 0.44 – 0.72 |  |  |  |  |
| **G.** | **Social isolation** | |  | **0.92** |  | **0.89 – 0.95** |  | **0.79** |  | **0.68 – 0.87** |
|  | 16 | Is rejected by other residents | 0.74 |  | 0.58 | 0.43 – 0.71 | 0.76 |  | 0.49 | 0.23 – 0.67 |
|  | 20 | Openly rejects contact with others | 0.70 |  | 0.61 | 0.50 – 0.70 | 0.68 |  | 0.42 | 0.22 – 0.58 |
|  | 32 | Calls out | 0.88 |  | 0.72 | 0.53 – 0.86 | 0.87 |  | 0.74 | 0.54 – 0.89 |
| **H.** | **Feeling at home** | |  | **0.87** |  | **0.82 – 0.92** |  |  |  |  |
|  | 13 | Indicates that he or she is bored | 0.73 |  | 0.45 | 0.29 – 0.59 |  |  |  |  |
|  | 28 | Indicates feeling locked up | 0.86 |  | 0.57 | 0.33 – 0.74 |  |  |  |  |
|  | 36 | Feels at home on the ward | 0.78 |  | 0.68 | 0.57 – 0.78 |  |  |  |  |
|  | 39 | Wants to get off the ward | 0.85 |  | 0.62 | 0.42 – 0.77 |  |  |  |  |
| **I.** | **Having something to do** | |  | **0.93** |  | **0.89 – 0.95** |  |  |  |  |
|  | 26 | Finds things to do without help from others | 0.86 |  | 0.80 | 0.70 – 0.88 |  |  |  |  |
|  | 38 | Enjoys helping with chores on the ward | 0.76 |  | 0.64 | 0.49 – 0.75 |  |  |  |  |
|  | **Remaining items to be used in future research** | |  |  |  |  |  |  |  |  |
|  | 9 | Does not want to eat | 0.70 |  | 0.56 | 0.43 – 0.67 | 0.70 |  | 0.59 | 0.43 – 0.72 |
|  | 15 | Enjoys meals | 0.86 |  | 0.72 | 0.60 – 0.82 | 0.76 |  | 0.59 | 0.40 – 0.75 |
|  | 30 | Likes to lie down (in bed) | 0.74 |  | 0.64 | 0.53 – 0.74 | 0.67 |  | 0.51 | 0.33 – 0.67 |

^a^ Overall proportion of agreement (the ratio of exact agreement between raters to the total number of ratings).

^b^ ICC: intra-class correlation coefficient.

^c^ Kappa values.
